# Microglial Extracellular Vesicles as Vehicles for Neurodegeneration Spreading

**DOI:** 10.3390/biom11060770

**Published:** 2021-05-21

**Authors:** Inês Dinis Aires, Teresa Ribeiro-Rodrigues, Raquel Boia, Magda Ferreira-Rodrigues, Henrique Girão, António Francisco Ambrósio, Ana Raquel Santiago

**Affiliations:** 1 Faculty of Medicine, Coimbra Institute for Clinical and Biomedical Research (iCBR), University of Coimbra, 3000-548 Coimbra, Portugal; inesaires9@gmail.com (I.D.A.); teresamrrodrigues@gmail.com (T.R.-R.); raquelfboia@gmail.com (R.B.); magdamfr96@gmail.com (M.F.-R.); hmgirao@fmed.uc.pt (H.G.); afambrosio@fmed.uc.pt (A.F.A.); 2 Center for Innovative Biomedicine and Biotechnology (CIBB), University of Coimbra, 3000-548 Coimbra, Portugal; 3 Clinical Academic Center of Coimbra (CACC), 3000-548 Coimbra, Portugal; 4 Association for Innovation and Biomedical Research on Light and Image (AIBILI), 3000-548 Coimbra, Portugal

**Keywords:** extracellular vesicles, exosomes, microglia, microvesicles, neurodegeneration

## Abstract

Microglial cells are the neuroimmune competent cells of the central nervous system. In the adult, microglia are responsible for screening the neuronal parenchyma searching for alterations in homeostasis. Chronic neuroinflammation plays a role in neurodegenerative disease. Indeed, microglia-mediated neuroinflammation is involved in the onset and progression of several disorders in the brain and retina. Microglial cell reactivity occurs in an orchestrated manner and propagates across the neural parenchyma spreading the neuroinflammatory signal from cell to cell. Extracellular vesicles are important vehicles of intercellular communication and act as message carriers across boundaries. Extracellular vesicles can be subdivided in several categories according to their cellular origin (apoptotic bodies, microvesicles and exosomes), each presenting, different but sometimes overlapping functions in cell communication. Mounting evidence suggests a role for extracellular vesicles in regulating microglial cell action. Herein, we explore the role of microglial extracellular vesicles as vehicles for cell communication and the mechanisms that trigger their release. In this review we covered the role of microglial extracellular vesicles, focusing on apoptotic bodies, microvesicles and exosomes, in the context of neurodegeneration and the impact of these vesicles derived from other cells in microglial cell reactivity.

## 1. Introduction

Microglial cells are the main neuroimmune cells of the central nervous system (CNS) that comprise 5–12% of the cells in brain [1] and 0.2% of total retinal cells [2]. In the adult brain and retina, microglia have three main essential functions: (i) sentinel function that involves a constant scanning of the environment with their highly dynamic processes, (ii) physiological housekeeping function that includes synaptic remodeling and migration to sites of neuronal death to phagocyte dead cells and (iii) protection against detrimental agents (reviewed in [3]). Despite their role on the maintenance of homeostasis, microglia are deeply involved in neuroinflammation, since a sustained response of microglia can have deleterious effects. Upon activation, microglia become highly mobile, decrease in size, retract their processes and release a plethora of factors that include inflammatory cytokines, chemokines and reactive oxygen species [3,4,5]. Together with all these soluble factors microglia also secrete extracellular vesicles (EVs) [6], which are involved in intercellular communication. Small EVs are capable of transmitting signals across tissue boundaries and of specifically targeting different cell types. In addition, EVs derived from other cells can also interact with microglial cells shaping their phenotype and function. Herein, we explore the role of microvesicles and exosomes released by microglial cells in neurodegeneration and also how the EVs shed by other cells may influence microglial cell function contributing to neurodegeneration. In this work, the distinction between microvesicles and exosomes was made based on the isolation methods and identification described in the original studies.

## 2. Contribution of Microglia to Neurodegeneration

In several neurodegenerative diseases, microglia become activated and change their transcriptional profile, morphology and function (reviewed in [7]). These activated microglia can perform different functions and exert beneficial or detrimental actions. Activated microglial cells produce and release neuroprotective factors, drive the inflammatory response in order to remove the damaged cells by phagocytosis and orchestrate neurorestorative processes [8]. The involvement of microglia and EVs has been explored in the context of several CNS disorders. Herein, we provide a comprehensive summary of the contribution of microglial cells to the conditions where EVs were shown to play a role, like traumatic brain injury, Alzheimer’s and Parkinson’s diseases, glaucoma and retinopathy of prematurity.

In the context of Alzheimer’s disease (AD), activated microglia are found surrounding pathological amyloid-β (Aβ) plaques [9], one of the major pathological hallmarks of this condition [10]. In experimental models, microglia also cluster around plaques [10], and the number and size of microglia increase in proportion to the size of the plaques [11,12]. The demonstration that microglia rapidly migrate toward new plaques [13,14] engulfing Aβ [14,15] suggests that activated microglia may be protective to some extent in the setting of Aβ pathology [9,13,14,15]. Moreover, the fact that microglial processes wrap around Aβ plaques acting as a physical barrier and preventing the outward extension of amyloid fibrils was identified as a protective function of microglia [16]. The gene TREM2 (triggering receptor expressed on myeloid cells 2) is highly expressed in microglia [17]. Several studies identified a mutation in TREM2 that is one of the genetic risk factors for developing AD [18,19], providing a clear link between microglia dysfunction and AD. The degree of neuronal loss in AD correlates with a downregulation of homeostatic microglial genes [20]. This loss of homeostatic microglial genes has been also observed in multiple sclerosis (MS) [21], which is a chronic inflammatory demyelinating disease of the CNS that progresses to profound neurodegeneration in the brain and spinal cord [22].

Despite different triggering events, neuroinflammation mediated by microglia is a common feature that drives progressive neuronal damage in multiple neurological disorders [23,24]. Although the initial activation of microglia is beneficial for clearing toxic Aβ from the brain in AD, over time the chronic stimulation of microglia by Aβ may also be deleterious and lead to prolonged inflammation, excessive Aβ deposition and acceleration of the neurodegenerative process [25].

Parkinson’s disease (PD) is characterized by a progressive dopaminergic neurodegeneration and Lewy bodies formation, which are formed by the aggregation of misfolded α-synuclein in the substantia nigra [26]. It was demonstrated that α-synuclein triggers microglial activation [27,28] and in vivo imaging and post-mortem immunohistochemical studies revealed the presence of activated microglia in the substantia nigra of PD patients [29,30,31,32]. Since several cytokines including interleukin (IL)-1β, IL-2, IL-4, IL-6, transforming growth factor-β and tumor necrosis factor (TNF) were found to be increased in the brains of PD patients [33], inflammation was proposed as a hallmark of PD [34]. Additionally, interferon-γ (IFN-γ), another critical inflammatory cytokine, plays a crucial role in microglial-mediated dopaminergic neurodegeneration [35]. Together these data suggest an important role for microglia in the pathogenesis of PD. In fact, microglia activation contributes to cognitive impairments in a rotenone-induced PD mouse model, and the depletion of microglia using pexidartinib (PLX3397), a tyrosine kinase inhibitor of colony stimulating factor 1 receptor that hinders microglia survival, or inactivation of reactive microglia with minocycline treatment ameliorates the cognitive deficits and neuronal damage [36].

Traumatic brain injury (TBI) leads to chronic and progressive neurodegenerative changes, being microglial-mediated neuroinflammation an important secondary injury mechanism [37]. Chronic inflammatory response has a preponderant role in TBI, since it was demonstrated that microglia activation can persist for months and years in experimental models and in humans that experienced TBI [37,38,39]. It was demonstrated that decreasing microglia activation, using pharmacologic or physical interventions, limits progressive neurodegeneration and improves neurological recovery [40,41,42]. Moreover, the depletion of microglia during the chronic phase of experimental TBI, with subsequent repopulation of microglia, reduces chronic neuroinflammation, improves neurological recovery (sensori-motor and cognitive function) and decreases neurodegeneration [43]. 

Microglia-mediated neuroinflammation also contributes to chronic and progressive neuronal loss in retinal degenerative diseases [5]. Indeed, microglia reactivity has been associated with the onset and progression of many ocular diseases like, glaucoma, retinopathy of prematurity, diabetic retinopathy and age-related macular degeneration [5,44]. Although with different etiologies, in all of these diseases microglial cells orchestrate a proinflammatory response that culminates in the loss of retinal neurons and dysfunction of the visual pathway [5,44,45]. Although microglia represent an important player in retinal diseases the role of microglia EVs in the retina is still scarcely explored, with studies in glaucoma and retinopathy of prematurity [46,47]. Glaucoma is currently largely recognized as a neurodegenerative disease [48,49], and is characterized by loss of retinal ganglion cells (RGCs), damage of their axons and optic nerve atrophy [50]. The immune system has received increasing attention, having a pivotal role in the initiation and propagation of the neurodegenerative process [5]. Microglia activation has a preponderant role in glaucomatous damage [45], and the control of microglia-mediated neuroinflammation was demonstrated to confer protection to RGCs [5,51,52]. The concept that microglia are central players in the pathophysiology of glaucoma arises with the observation that microglia activation occurs earlier than RGC loss and even before elevation of intraocular pressure, the main risk factor for glaucoma onset and progression, in the DBA/2J mouse model of glaucoma [53].

Retinopathy of prematurity is a neurovascular complication leading to childhood blindness [54]. This disease is characterized by initial arrested retinal vascularization followed by neovascularization and subsequent retinal detachment causing permanent visual loss [55]. The complement system is implicated in the disease. Complement activation regulates microglia/macrophage activation in the retina and vitreous that creates an imbalance in angiogenic and antiangiogenic factors [56]. The understanding of how microglia communicate through EVs and the mechanisms that are able to control their response is of the utmost importance to tackle neurodegenerative diseases.

## 3. Extracellular Vesicles

The maintenance of the homeostasis, integrity and proper function of an organism or system relies in a fine-tuned communication between the different cells by which it is composed. Among these conveyers of information are EVs, membrane-enclosed nanoscale particles, released by virtually all prokaryotic and eukaryotic cells as part of their normal physiology and in response to injury, being associated with tissue/organ remodeling and implicated in pathology development. Although initially perceived as innocuous packages of cell “trash”, in the last years EVs have gained particular attention as players of a more orchestrated and comprehensive strategy of a cell response to internal and external cues [57]. Indeed, EVs are now consistently considered as a way of the cells producing them to modify their molecular landscape and get rid of unnecessary or unwanted constituents. Production of EVs helps to maintain cellular homeostasis, and more importantly, upon release EVs act as crucial vehicles for long distance information exchange between cells, thus assisting in tissues and organs crosstalk. EVs have been associated with viral pathogenicity/spreading, immune responses, cancer progression, neuronal and cardiovascular diseases [58,59,60,61,62,63,64]. Depending on their origin and mechanism of biogenesis, EVs can carry multiple cell constituents, including nucleic acids namely mRNA, noncoding RNA species and DNA, metabolites, proteins and lipids [65,66,67]. Moreover, the presence in easily accessible biological fluids make EVs powerful clinical tools to help in disease diagnosis and prognosis [68,69]. Since they can not only mirror the pathophysiological state of disease-causing cells but also the general physiological state of the organisms. EVs have also been considered as promising therapeutic vehicles for many diseases, including cancer and neurodegenerative diseases [70,71].

**Figure 1:**
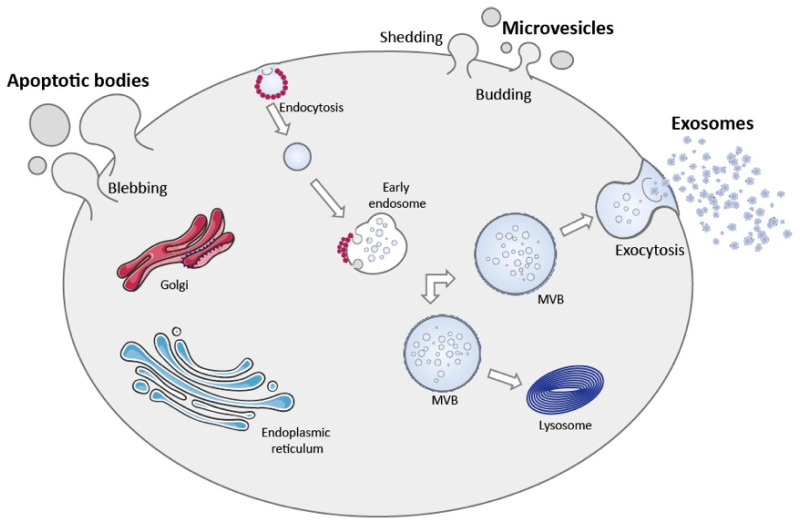
Schematic representation of the mechanisms involved in EV biogenesis and release. Microvesicles are shed into the extracellular space by direct budding of the plasma membrane. Apoptotic bodies are released by membrane blebbing of apoptotic cells upon cell disintegration. Exosomes are small EVs derived from the endocytic pathway that are released into the extracellular milieu by the fusion of the MVBs with the plasma membrane. During their formation MVBs can either release their content into the extracellular space through exocytosis or alternatively fuse with lysosomes leading to vesicle degradation.

Due to the difficulty in assigning an EV to a particular biogenesis pathway, mainly due to the lack of specific markers of EV subtypes, the International Society for Extracellular Vesicles (ISEV) has established guidelines to isolate and classify different types of EVs and non-vesicular nanoparticles like exomeres [72]. Accordingly, it is suggested that EVs should be referred using operational terms like descriptions of conditions or cell of origin, biochemical composition or physical characteristics such as size [73]. However, in general terms and according to their biogenesis, EVs can be divided into two main categories: exosomes and ectosomes or shedding vesicles (Figure 1).

Exosomes are small EVs, with a size range from approximately 40 to 160 nm in diameter, formed by the inward budding of the endosomal membrane, which gives origin to intraluminal vesicles (ILVs), during the maturation of the multivesicular bodies (MVBs), and released upon fusion of the MVBs with the plasma membrane (Figure 1) [74]. Different mechanisms and players have been implicated in exosomes biogenesis, namely Rab GTPases, subunits of endosomal sorting complex required for transport (ESCRT), syntetin-1, tumor susceptibility gene 101 (TSG101), apoptosis-linked gene 2-interacting protein X (ALIX), ceramide, sphingomyelinases and tetraspanins including cluster of differentiation (CD) 9, CD63 and CD81, which also regulate cargo sorting into the vesicles [75,76,77,78]. Exosome formation, either in an ESCRT-dependent or independent way, is a complex process that varies according to the cell type and may be influenced by intracellular and extracellular signals, modulating final vesicle cargo and release, which ultimately impacts intercellular communication.

On the other hand, ectosomes are vesicles that originate from the outward budding of the plasma membrane, with a size range from 50 nm to 1 µm in diameter and include microvesicles and apoptotic bodies among others (Figure 1). The mechanisms and players that regulate microvesicles released by healthy cells have just started to be unraveled. However, similarly to exosomes, the formation of microvesicles partially depends on ESCRT proteins and the generation of ceramide by sphingomyelinase. Additionally, microvesicles biogenesis requires reorganization within the plasma membrane of protein and lipid components, including flipping of phosphatidylserine from the inner leaflet to the cell surface with consequent physical bending of the membrane and reorganization of actin cytoskeleton, culminating in membrane budding and vesicle release [74,79].

It is worth mentioning that the content profile (proteins, nucleic acids and lipids) of EVs does not merely directly reflects the changes in the cells of origin. Supporting the idea that EVs content is selectively sorted, a process that is influenced by external conditions affecting the producing cell. This will induce specific responses on recipient cells and participate in the regulation of processes like receptor–ligand signaling, immune response and regulation of central and peripheral immunity, angiogenesis, cellular differentiation and neoplasia and metabolic reprogramming, among others [67,80,81].

Once into the extracellular space, EVs can reach recipient cells and potentially promote a cellular response according to their cargo landscape. After docking to the recipient cell, EVs can remain bound to the surface and trigger intracellular signaling pathways through the activation of receptors on the recipient cell, or be internalized [76]. EVs internalization can occur by different mechanisms, including clathrin-mediated or clathrin-independent endocytosis (macropinocytosis and phagocytosis), caveolae and lipid raft-mediated endocytosis. After uptake by different mechanisms, EVs follow the endocytic pathway, and if not targeted and consequently degraded by the lysosome, they release their content into the cytoplasm [82,83,84]. Additionally, EVs can also fuse directly with the plasma membrane or endocytic membrane (post internalization) of recipient cells, enabling the exchange of transmembrane proteins and lipids, and delivering the EVs cargo, including mRNA and miRNAs, into the target cell, regulating gene expression and triggering a cell response, changing cell phenotype and modifying its biological function [85]. Alternatively, vesicles can return to the extracellular space (transcytosis) by endosome fusion with the plasma membrane [57]. More recently, a landmark study showed that connexin 43 (Cx43), a gap junction protein, can mediate the release of EVs content into recipient cells [86]. However, the mechanisms that facilitate vesicle interaction with the target cell, thus conferring cell or organ tropism to EVs remain largely elusive [87,88,89,90]. A clarification of these mechanisms, combined with intrinsic properties of EVs as regulators of intracellular pathways and the possibility to engineer them to deliver diverse agents, foresees an important role for EVs as therapeutic agents in a near future [91,92,93,94,95,96,97]. Moreover, their presence in biological fluids and easy accessibility may aid in diagnosis and monitoring of disease progression and confirming response to therapy.

### 3.1. Apoptotic Bodies

Apoptosis is a highly regulated form of cell death that occurs as part of development, physiological tissue remodeling and function or as a consequence of cell damage [98]. There are many signals involved in the triggering of apoptotic cell death that may vary from cell to cell [98,99]. Apoptotic bodies are released by cells undergoing apoptosis, formed by membrane enclosed vesicles that contain cellular cargo from disintegrating cells (see Figure 1). Programmed cell death or apoptosis is mediated by the activation of caspases, proteolytic enzymes that exist in every cell in its inactive form called procaspases [99]. Upon activation, caspases signal to downstream effectors that will degrade intracellular compartments in an organized manner [99]. The formation of apoptotic bodies is initiated by generation of membrane blebs through the coordinated action of several protein kinases namely caspase-activated Rho-associated kinase 1 (ROCK1) [100,101,102]. These blebs then extend radially forming long protrusions with engulfed cellular components. The bleb elongation process is regulated by other caspase-activated proteins, caspase-activated membrane channel pannexin 1 (PANX1) that negatively regulates bleb formation or in some cell types caspase-cleaved membrane receptor plexin B2 (PLXB2) that favors bleb elongation [103,104]. The disassembly of PANX1 promotes the release of apoptotic bodies from the dying cell through the fragmentation of the cellular structure [105]. Apoptotic bodies present a variable composition and can encompass a variety of cellular structures like, micronuclei, chromatin residues, portions of the cytosol, protein aggregates, DNA fragments or even organelles [99,106]. The loading of apoptotic vesicles was thought to be a dysregulated process occurring in a fortuitous manner [99]. Nevertheless, the recent finding that these vesicles are selectively endowed with either RNA or DNA may suggest otherwise, posing that apoptotic bodies may present different functional properties [107]. These vesicles are limited by a lipidic bilayer, where phosphatidylserine residues are exposed at the outer leaflet, acting as a “eat me” signal for phagocytic cells [99]. This conveys a “clean” cell death process where cell debris are efficiently cleared from the parenchyma without eliciting an inflammatory response or major alterations in homeostasis [99]. Although apoptotic bodies may be regarded as waste containers some important functions have been attributed to these vesicles [108]. As other EVs, apoptotic bodies were shown to mediate cell communication by transferring biomolecules between cells that are capable of modulating recipient cell function. For instance, apoptotic bodies facilitate the horizontal gene transfer between neighboring cells, by encapsulating genomic DNA fragments [109,110]. Furthermore, these vesicles were shown to be involved in endothelial cell communication, transferring miR-126 to healthy endothelial cells and altering their function, inducing a feedback loop that allowed the recruitment of progenitor cells hindering disease progression [111]. Apoptotic bodies also play a central role in immune regulation, by transferring autoantigens and proinflammatory molecules [99]. Neuronal apoptosis is a hallmark of several CNS disorders, rendering signaling disfunction and accumulation of apoptotic material [112]. Microglia were shown to actively clear cellular debris upon cell death, namely apoptotic bodies (reviewed in [113,114]). The role of apoptotic bodies in mediating microglial cell function is still scarcely explored. In most circumstances microglial cells efficiently degrade apoptotic bodies, being their effects mediated by upstream signaling occurring prior vesicle engulfment [115]. Indeed, microglial cells respond to a multitude of signals emitted by distressed cells that allow their recognition [116,117]. This clearing task of microglial cells is modulated by specific residues presented at the surface of apoptotic bodies, like phosphatidyl serine, that are recognized by receptors present in microglial cells [118,119]. The engulfment of apoptotic material by microglia is usually inert or even protective [117,120], although the accumulation of undigested vesicles may lead to cell dysfunction and proinflammatory signaling [115]. On the other hand, overactivation of microglia leads to microglia apoptosis [121]. This was proposed to occur in an attempt to self-limit a proinflammatory deleterious response [121].

### 3.2. Microvesicles

Microvesicles are EVs commonly released by cells, formed by the outward budding of the plasma membrane. The precise mechanisms of microvesicle formation are still poorly understood, however there is consensus in the field that their formation and release are highly regulated processes involving the selective loading of cargo [122]. The biogenesis of microvesicles requires localized molecular changes within the plasma membrane that decrease rigidity and allow the curvature of the membrane, achieved through phospholipid and cytoskeletal protein rearrangement [123]. This process is initiated by the formation of membrane microdomains by lipids and membrane associated proteins, and the recruitment of ESCRT-I that selects cellular components, like proteins and RNA, to be released in microvesicles [74,122]. Following this step, the translocation of phosphatidylserine residues to the outer leaflet of the plasma membrane initiates the formation of a membrane bleb by promoting the physical bending of the plasma membrane and restructuring of the actin cytoskeleton [74]. This is achieved through the action of aminophospholipid translocases (flippases and floppases), scramblases and calpain, proteins that promote the switching of phospholipids from the outer to the inner leaflet of the plasma membrane [74,124]. However, studies conducted in endothelial cells and platelets demonstrated that in the absence of phosphatidylserine translocation and with the integrity of the plasma membrane maintained, microvesicles are still shed [125,126]. This finding suggests that other mechanisms might be involved in microvesicles formation. Alterations in cholesterol composition of the plasma membrane that promote membrane fluidity were also proposed to contribute to microvesicles biogenesis. Indeed, it was shown that the depletion of cholesterol from neutrophils hinders microvesicle formation, showing its central role in the formation of membrane microdomains [127]. Following budding formation microvesicles are released into the extracellular space by alterations in the cytoskeleton that actively pinch the newly formed bleb out of the cell. The restructuring of the cell cytoskeleton is caused by actin–myosin interactions. Myosin contracts due to its phosphorylation by activated GTPase adenosine diphosphate (ADP)-ribosylation factors (ARF6 and ARF1), which leads to the budding of the microvesicle [79,122]. The released microvesicles may vary in size from 50 to 1000 nm, while larger microvesicles have also been detected. After release, microvesicles can bind to recipient cells through receptor interaction, fusion or be endocytosed. Similar to what was described for apoptotic bodies, exposed phosphatidylserine residues in the outer membrane of microvesicles are recognized by receptors for phosphatidylserine and can shift recipient cells response following internalization via fusion or endocytosis [128]. 

### 3.3. Exosomes

The biogenesis of exosomes is dependent of diverse mechanisms and players. Considering that these vesicles originate in the endolysosomal pathway, its dependence on ESCRT machinery is not surprising. The ESCRT machinery is composed by multiprotein complexes and additional associated proteins, like ALIX and vacuolar protein 4 (Vps4), which associate in a stepwise manner at the endosome membrane, regulating the cargo selection on microdomains (ESCRT-0 and -I) and the formation of ILVs by budding and fission of this microdomains (ESCRT-II and -III). This complex machinery recognizes the pattern of ubiquitinated proteins in the cell using the ESCRT-0, initiating the MVB pathway. Following this, ESCRT-0 recruits ESCRT-I through the connection of HRS (hepatocyte growth factor-regulated tyrosine kinase substrate) PSAP domains to the TSG101 subunit, helping to recognize targeted proteins [77]. Impairment of ESCRT-0 or -I by depletion of complexes that recognize ubiquitin patterns like HRS, STAM1 (signal transducing adapter molecule 1) or TSG101 results in a decreased secretion of exosomes [77,129]. Following the recognition of ubiquitinated proteins ESCRT-I promotes ESCRT-II activity that is involved in the formation of a nucleation point, and the subsequent recruitment and activation of the ESCRT-III protein complex, lastly promoting vesicle budding [77]. The formation of vesicles is stabilized by the adaptor protein ALIX. Indeed, syntetin bound to syndecan endosomal microdomains, whose formation is facilitated by heparanases, recruits the ESCRT adaptor protein ALIX and consequently ESCRT-III machinery, supporting membrane budding and leading to ILV formation [78,130]. The end of the MVB pathway is determined by the disconnection and recycling of the ESCRT-III complex from the endosomal membrane, promoted by the ATPase Vps4 complex [131,132]. The MVBs can then fuse with the plasma membrane or with the lysosome for degradation.

Alternatively, MVB biogenesis and consequent exosome formation can also occur through ESCRT-independent processes [133]. Within distinct small subdomains on the endosomal membrane, sphingolipids namely sphingomyelin, are converted by neutral sphingomyelinases, to phosphorylcholine and ceramide. The presence of ceramide, a cone-shaped rigid lipid, induces coalescence of these subdomains into larger domains promoting budding and ILV formation [134,135,136]. Tetraspanins are four-transmembrane domain proteins that were described to select cargo for ILVs in a mechanism independent of ESCRT and ceramide. In addition, heat shock proteins (HSP), such as the chaperone HSP70 were found to recognize specific motifs in cytosolic proteins recruiting them to ILVs [137]. It is unknown whether a MVB is produced by a single mechanism or if the accumulation of ILVs can be afforded by different machinery, promoting heterogeneity in the exosomes released. The different mechanisms involved in exosome biogenesis may differ according to the cell type, stimulus or the cargo, reinforcing the idea that cells secrete a heterogeneous population of vesicles that reflect different types of MVBs or different pathways of ILV formation within the same organelle. 

## 4. Microglial Microvesicles in Neurodegeneration

**Figure 2:**
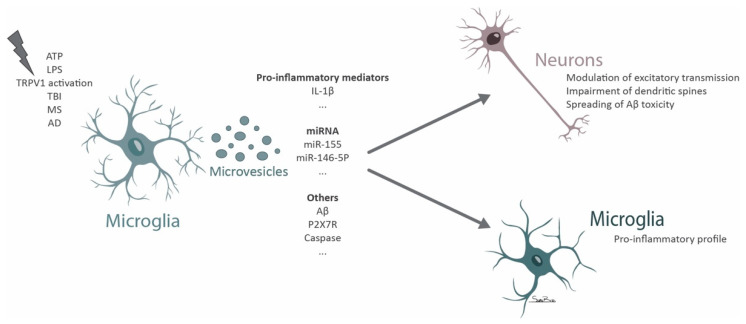
Scheme summarizing the role of microglial microvesicles in neurodegeneration.

Microvesicles may be an important route of glial communication (Figure 2). Indeed, when subjected to mechanical stress, astrocytes release large amounts of adenosine triphosphate (ATP) that influence microglia function [138], inducing the shed of microvesicles in microglia, dependent on the activation of purinoreceptors like P2X7 receptors (P2X7R) [138]. These microvesicles are endowed with caspase-1 (both proenzyme and active forms) and IL-1β, suggesting that microvesicles may host enzymatic activity to deliver the active form of IL-1β [138]. Microvesicles released upon ATP stimulation also contain P2X7R, which might serve as a protective mechanism against apoptosis, avoiding the deleterious stimulation by ATP [138]. Microvesicle shedding may account for an important pathway for IL-1β secretion by microglial cells, allowing long-distance effects in receptor cells [138]. Indeed, it was described that upon spinal nerve ligation microglia release microvesicles enriched in IL-1β through the activation of the P2X7R-p38 pathway [139]. These vesicles were shown to contribute to neuropathic pain by decreasing paw withdrawal threshold and paw withdrawal latency [139]. Microglia express the transient receptor potential vanilloid type 1 channel (TRPV1) [140] that is associated with neuropathic pain [141]. The activation of this receptor with capsaicin was shown to cause microglia activation and microvesicle shedding that enhanced neuronal glutamatergic transmission [141], indicating that this may represent another pathway in the communication between microglia and neurons.

Microglia isolated from TBI mice were shown to release microvesicles that can induce microglia reactivity in vitro [142]. When microglial cells in culture are exposed to lipopolysaccharide (LPS) the release of microvesicles enriched in IL-1β and miR-155 increases [142], providing hints for the mechanism responsible for the progression of neuroinflammation. Furthermore, the cortical injection of microvesicles derived from microglia isolated from the cortex of mice subjected to TBI or treated with LPS leads to the activation of microglia in the brain of naïve animals [142]. These observations suggest a mechanism of autologous communication between microglia through microvesicles, that upon a deleterious stimulus can further contribute to the dispersion of the neuroinflammatory signal [142]. Interestingly, the activation of microglia with LPS seems to increase not only the number but also the size of the shed vesicles [143].

Microglia release microvesicles that can be detected in the cerebrospinal fluid (CSF) of healthy controls and seem to be increased in patients with multiple sclerosis [144]. In a mouse model of multiple sclerosis (experimental autoimmune encephalomyelitis), microvesicles originated from microglia/macrophages were reported to spread the inflammatory environment, while the impairment of vesicle shedding was protective [144]. To reinforce the role of microvesicles in disease progression, the administration of FTY720, a drug that was shown to be beneficial in multiple sclerosis, decreases the presence of microvesicles in the CSF of mice with experimental autoimmune encephalomyelitis [144].

Microglia microvesicles were found to be increased in the CSF of patients with mild cognitive impairment and AD [145,146]. These vesicles were toxic to neurons following in vitro exposure [145] and were also found to be detrimental in patients being myelinotoxic and neurotoxic in the presence of Aβ [146]. The soluble form of prefibrillar Aβ species is the most toxic form of Aβ [147]. The authors describe that the neurotoxicity induced by microglia microvesicles is due to the increased solubility of Aβ peptides when associated with vesicle lipids and to the spreading of Aβ promoted by microvesicles [145]. In addition, it was described that the levels of microglial microvesicles present in the CSF correlate with the atrophy of the hippocampus in AD and with white matter tract alterations detected by MRI in patients with mild cognitive impairment [146].

Proinflammatory microglia release microvesicles enriched in miRNAs that control the expression of synaptic proteins [148]. One relevant miRNA that was identified was miR-146a-5p, a miRNA specific of microglial cells (not expressed by neurons), that is transferred by microglial microvesicles and decreases excitatory synapses due to its detrimental effect in dendritic spines [148]. miR-146a-5p suppresses presynaptic synaptotagmin-1 and postsynaptic neuroligin-1, proteins that play a crucial role in dendritic spine stability [148]. Inhibiting the contact of microvesicles with recipient neurons by cloaking phosphatidylserine residues or depleting miR-146a-5p from the vesicles among other strategies that impaired the miRNA transfer, sustains the function of neuronal synapses [148]. In addition, microglia shed microvesicles that are able to increase neuronal excitatory transmission by promoting the production of ceramide and sphingosine, increasing synaptic vesicle release by the facilitation of SNARE (soluble NSF (N-ethylmaleimide-sensitive factor)) complex formation [149]. Microglia microvesicles were shown to induce glutamate release and promoting a dose dependent increase in miniature excitatory postsynaptic current frequency and an increase in the amplitude of evoked visual field potentials in the visual cortex [149]. Microglial cells were also shown to modulate presynaptic transmission through the modulation of the endocannabinoid system by releasing microvesicles containing endocannabinoid N-arachidonoylethanolamine on their surface [150]. These vesicles can activate type-1 cannabinoid receptors in GABAergic neurons inhibiting presynaptic transmission [150].

## 5. Microglial Exosomes in Neurodegeneration

**Figure 3:**
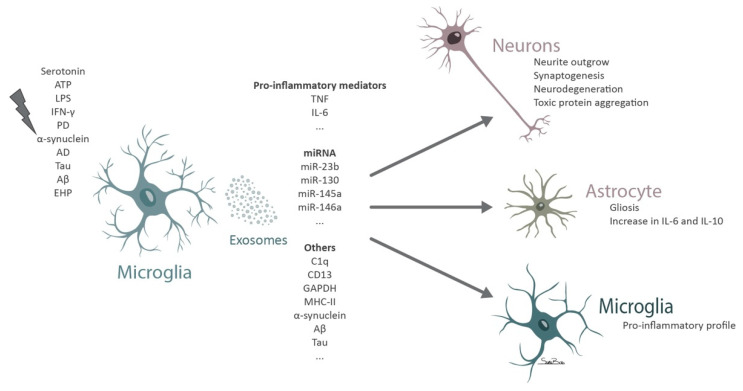
Diagram depicting the contribution of microglial exosomes to neurodegeneration.

The release of exosomes by microglial cells can be stimulated by various signals besides the constitutive release occurring in homeostasis (Figure 3).

Under physiological conditions the release of serotonin by neurons induces the release of exosomes by microglial cells through the activation of 5-HT2a,b and 5-HT4 serotonin receptors, due to an increase in intracellular calcium levels that promote the fusion of the MVBs with the plasma membrane [151]. Other studies reflect the content of constitutively released microglia exosomes. The protein content of these vesicles seems to reflect that of the parental cells, enriched in microglia-specific proteins, such as proteins responsible for maintaining microglia quiescent state, synaptic pruning and anti-inflammatory molecules [152]. In addition, it was suggested that microglial exosomes can actively move without the need for extrinsic transport or fluidic movement as they contain several proteins, like Slingshot protein phosphatase 3, Coronin-1A, Cofilin-1, Plectin, Tropomodulin 2 and WAS/WASL-interacting protein family member 1, that control the reorganization and dynamics of actin filaments [151,152]. Exosomes released from microglial cells were shown to selectively express complement component 1q (C1q) subunits [152], CD13 and lactate transporter monocarboxylate transporter 1, since these proteins were not previously described in EVs [153]. These proteins identified in microglial exosomes might be useful to distinguish this population from other vesicles. Exosomes derived from microglia upon toxic exposure to alcohol promote C1q mediated neuronal cell death [154]. Interestingly, a recent report demonstrated that the glia–neuron communication mediated by EVs is maintained even between species [155]. The authors isolated exosomes from leach microglial cells and observed an increase in neurite outgrowth in rat primary neurons [155].

Several reports demonstrate increased production and release of exosomes upon stimuli, such as an increase in extracellular ATP, IFN-γ or exposure to LPS [152,156,157]. Exposure of microglia to ATP induces the release of exosomes enriched in proteins involved in cellular metabolism, in the autophagy-lysosomal pathway, cell adhesion and extracellular matrix organization [152]. These exosomes do not necessarily induce a negative response in astrocytes since an increase in proinflammatory IL-6 but also anti-inflammatory IL-10 expression occurs [152]. In addition, exosomes derived from ATP stimulated microglia are enriched in proteins that promote neurite outgrowth and synaptogenesis and others that negatively regulate neuronal apoptosis, suggesting that these exosomes have the potential to protect neurons [152]. On the other hand, exosomes released by microglia that were exposed to ATP seem to reflect the activation state of the parental cells and contribute to spread the inflammatory milieu, by activating astrocytes [152]. Similar to what was demonstrated for microvesicles, the activation of P2X7R in microglial cells leads to the release of exosomes [158,159]. Indeed, the oral administration of an inhibitor of P2X7R in a mouse model of AD promoted an improvement in working and contextual memory, while increasing the accumulation of exosomal proteins in microglial cells [158]. This suggests that hindering exosome release triggered by the activation of P2Y7R in microglial cells could afford protection against tauopathy, as confirmed by in vitro experiments [158].

When microglial cells are exposed to LPS, the number of secreted vesicles and the levels of proinflammatory cytokines, like TNF and IL-6, increase [143]. Indeed, the inhibition of TNF with etanercept is capable of decreasing the release of vesicles by microglia and also modify their content [143]. Proteome analysis of microglia EVs after exposure to LPS reveals an increase in proteins associated with translation and transcription, with a notorious difference for the proteome of control microglia exosomes in the presence of inflammatory mediators [143].

After priming with LPS, microglia respond to alterations in extracellular ATP through the activation of P2X7R and secretion of exosomes containing glyceraldehyde-3-phosphate dehydrogenase (GAPDH), which might be involved in the regulation of neuroinflammation and neurite formation in the brain [157]. Incubation of midbrain slice cultures with IFN-γ and LPS promotes the activation of microglia and an increase in the release of exosomes that induce dopaminergic degeneration [156]. The authors suggested that this pathway for neuronal degeneration may resemble those of PD where there is a progressive degeneration of dopamine neurons mainly mediated by microglia activation [156]. In addition, the exposure of microglia to α-synuclein promotes microglia activation and an increased release of exosomes with high levels of major histocompatibility complex class II and TNF, inducing neuronal cell death [160].

Several reports have associated exosomes to the spreading of neurodegeneration in models of AD [161,162,163,164,165,166] and PD [167,168,169]. Although, with different etiologies, both diseases share some similarities, as abnormal deposition of proteins that form aggregates that hinder normal neuronal function [170]. Protein aggregates of Aβ, tau and α-synuclein were identified in exosomes isolated from the CSF of patients with AD and PD [171]. Microglial cells phagocyte α-synuclein aggregates leading to the incorporation of fibrils into exosomes [172]. These vesicles, accompanied by a neuroinflammatory environment induce protein aggregation in recipient neurons [172]. The stereotaxic injection of microglial exosomes containing α-synuclein, promote the spreading of aggregates along several brain regions [172]. The depletion of resident microglia decreases the transmission of α-synuclein [172], indicating the critical contribution of microglia and microglia-derived exosomes in the pathology. In this report, exosomes containing CD11b and α-synuclein were detected in the CSF of PD patients, probably derived from microglial cells [172].

AD is characterized by the presence of neurofibrillary tangles formed by abnormally phosphorylated tau protein [173]. This altered tau protein was shown to work as a prion-like identity being able to recruit native tau into the formation of fibrillary aggregates [174]. The transmission of these aggregates between cells was proposed to be an attempt for cells to efficiently eliminate them upon an overwhelming of the proteostasis endogenous system [175,176]. The release of neurotoxic proteins in exosomes would allow cells to shuttle the protein aggregates to other cells with a possible better degradation capacity. Indeed, into a limited extension microglia were shown to degrade phagocyted aggregates through the action of lysosomes [87]. Furthermore, tau lacks a known secretory pathway, the established route for tau pathology spread was due to cell death that spreads tau protein through cell disintegration [174,177]. Exosomes have recently arisen as a vehicle for tau propagation [178]. Exosomes containing tau and bridging integrator 1 (BIN1) were identified in the CSF of patients with AD [179]. The overexpression of BIN1 was associated with the release of exosomes containing tau by microglia and a worsening of the disease in a mouse model of AD [179]. Interestingly, this effect was blocked, mainly in males, by genetic deletion of BIN1 that promoted a reduction in the expression of HSPs involved in tau proteostasis [179]. This suggests a sex-specific role for BIN1 in the regulation of tau proteostasis and secretion through exosomes in microglia [179]. Indeed, microglia were shown to selectively uptake tau and to release and spread the neurotoxic stimulus by incorporation tau protein in exosomes and transmitting it across the brain parenchyma [161]. In line with these findings, it was demonstrated that either the inhibition of exosome synthesis [161] or the depletion of microglia was able to decrease tau propagation in the brain of a mouse model of AD [161]. A recent study further supported the role of microglia in the spreading of tau in a humanized amyloid precursor protein mouse model, by uncovering that microglia hypersecrete EVs that contribute to plaque deposition, while depleting microglia halted this effect [180]. In accordance, the decrease in exosome secretion through the inhibition of neutral sphingomyelinase-2, decreases Aβ plaque formation in a mouse model of AD [181]. Interestingly, microglia were shown to release exosomes containing a zinc metallopeptidase, known as the insulin degrading enzyme, which efficiently degrades Aβ [182]. The secretion of exosomes enriched in the insulin degrading enzyme is highly induced by statins, a class of drugs that decreases the risk of developing AD [182], suggesting that modifying the content of microglial exosomes may confer protection. Several reports show that an increase in exosomal release is accompanied by microglia reactivity [6,143,160]. Opposing to this it was shown that the exposure of microglia to Wnt3a, member of a family of cysteine rich glycoproteins that are implicated in the pathogenesis of AD, stimulates the release of exosomes by microglia without inducing a proinflammatory or neurotoxic profile in these cells [183]. The exosomes released by microglia exposed to Wnt3a were mainly enriched in proteins essential for structure, metabolism and proteostasis, and those include β-actin, GAPDH, ribosomal subunits and ubiquitin [183]. Overall, the data gathered showed a common mechanism for neurodegenerative disorders propagation, involving the spreading of dysfunctional proteins by microglial cells through exosomes. The impairment of exosome secretion might provide a new therapeutic approach to halt the degenerative process and ameliorate patient outcome. 

Previous reports determined an increase in the levels of glutaminase 1 in chronic CNS pathologies and in acute brain injury [184,185,186,187]. This mitochondrial enzyme is involved in the synthesis of glutamate through the hydrolysis of glutamine [188]. The exposure of microglia to elevated levels of glutaminase 1 increase the release of exosomes that are enriched in proinflammatory miRNAs (namely miR-23b, miR-130, miR-145a and miR-146a) [184]. Recently, in an in vitro model of ischemic stroke, microglial cells were shown to release exosomes enriched in miR-424-5p [189]. These vesicles further aggravate microvascular endothelial cell dysfunction upon oxygen-glucose deprivation, by regulating the FGF2/STAT3 pathway [189]. Using a mouse model of middle cerebral artery occlusion, the authors revealed that the inhibition of miR-424-5p reduced neurological and endothelial dysfunction [189], suggesting a role for microglial exosomes in stroke pathology.

Despite cumulating evidence for the role of microglial exosomes in the brain little is known regarding the exosomes derived from microglial cells in the retina. In a model of retinopathy of prematurity microglial exosomes were shown to confer protection against disease progression by decreasing pathological neovascularization and photoreceptor cell death [47]. Recently we demonstrated that retinal microglia release exosomes that contribute to amplify retinal neuroinflammation [46]. In glaucoma, a major cause of blindness worldwide and characterized by loss of retinal ganglion cells [190], it is widely recognized the contribution of microglia to glaucomatous neurodegeneration [5]. When microglial cells are challenged with elevated hydrostatic pressure (EHP), to mimic ocular hypertension [52], the cells become reactive and release proinflammatory mediators [5,51,52,191,192]. Moreover, these reactive microglia release exosomes that code for an inflammatory response [46]. These vesicles cause additional microglial reactivity, as determined by increased migration and phagocytosis, and trigger the release of proinflammatory cytokines that culminate in retinal cell death [46]. The exosomes derived from reactive microglia may be seen as a mechanism to spread the inflammatory signal between microglia or with other cell types, orchestrating the death of RGCs.

## 6. Extracellular Vesicles Derived from Other Cells and Their Role in Microglia-Mediated Neurodegeneration

As mentioned above, putatively all cells have the capacity to release EVs. Therefore, besides the effects of microglial cell EVs on other cells, the EVs found in the parenchyma released by other cells also shape the phenotype of microglial cells.

Microglial cells, as main phagocytes of the brain parenchyma, actively phagocyte Aβ and exosomes released by neuronal cells enriched in Aβ [163,193]. Both the exposure of mixed brain cultures and the intracerebrally injection of exosomes showed that these vesicles are preferentially uptaken by microglia [87,194], most likely due to the presence of phosphatidylserine in the membrane of exosomes that is recognized by phosphatidylserine receptors present at the microglia, commonly associated with the recognition of dead cells [193,195]. However, it is still under discussion whether the expression of phosphatidylserine residues in the membrane of exosomes would not lead to their quick degradation [196]. The uptake of neuronal exosomes containing Aβ promotes a shift in microglial cell response and as mentioned above contributes to the spreading of neurodegeneration. In addition, exosomes seem to influence the progression of motor neuron diseases by shifting microglial cells phenotype [197]. The expression of mutant copper-zinc superoxide dismutase 1 in motor neurons (mSOD1) (G93A) promotes the release of exosomes enriched in miR-124 [198]. These vesicles are uptaken by microglia and severely compromise their function by decreasing their phagocytic capacity and inducing an orchestrated inflammatory response [198]. 

Furthermore, exosomes have been described as important vehicles for neuron–glia communication. Indeed, it was found that cortical neurons release exosomes upon potassium-induced depolarization by an increased calcium influx that promotes the fusion of the MVB with the plasma membrane [199]. These exosomes were proposed to have a regulatory function of synapses allowing cell-to-cell exchange of proteins like adhesion molecules, prion proteins and subunits of glutamate receptors [199]. Following depolarization, neuronal exosomes were shown to induce synaptic pruning through the upregulation of complement molecule C3 in microglial cells [200]. Microglia receive oligodendrocytes exosomes through micropinocytosis and physiologically degrade the transferred material by trafficking it to the lysosomes [87]. This seems to be an efficient way to degrade oligodendrocyte membranes without inducing an immunological response, since these exosomes do not change microglia profile by inducing cell motility or cytokine secretion [87].

Besides their constitutive function, EVs also participate in the formation of metastatic niches and favor tumor growth and formation [201,202]. In the brain, glioblastoma EVs were shown to modulate microglia cell function to favor tumor development by enhancing the production of membrane type 1-matrix metalloproteinase by these cells [203]. In addition, in vivo experiments provided evidence that microglia uptake glioblastoma EVs, which are enriched in miR-451 and miR-21 that effectively downregulate the expression of their target c-Myc in microglia [204]. EVs may function as a mechanism for intercellular communication favoring tumor formation and spreading along distant brain regions by modulating a local immune response.

Protective roles of EVs have also been described, acting namely through the modulation of microglial cell response. Indeed, exosomes derived from hypoxia-preconditioned mesenchymal stromal cells can rescue cognitive decline in a mouse model of AD. These exosomes interact with microglial cells and regulate miR-21 ameliorating synaptic function, decreasing Aβ accumulation and inflammatory response [205]. Furthermore, in a model of TBI, the administration of exosomes from human stem cells of odontogenic origin was shown to decrease neuroinflammation in vivo and to prevent microglia response in vitro [206]. 

Opposite to what was described above for microvesicles derived from microglia isolated from TBI brains, exposure of microglial cells to neuronal extracts from TBI brains lead to the release of anti-inflammatory and neurotrophic exosomes enriched in miR-124-3p, from microglia in vitro promoting neurite outgrowth [207]. 

Peripheral inflammation can also reach the CNS through exosomes. After the intraperitoneal injection of LPS, serum exosomes enriched in miR-15a, miR-15b, miR-21 and miR-155, cross the blood brain–barrier and induce microgliosis, with increased levels of miR-155 and proinflammatory cytokines (TNF and IL-6) [208]. Interestingly upon peripheral inflammation, there was an increase in the presence of MVBs in the epithelium of the choroid plexus, which was accompanied by an increase in the presence of EVs in the CSF [209]. These vesicles were enriched in proinflammatory miRNAs like, miR-146a and miR-155 [209]. In vitro experiments using choroid plexus epithelium showed that upon LPS stimulation these cells release EVs that after injection into the lateral ventricles of naïve mice are taken up by brain cells, namely by astrocytes and microglia eliciting a proinflammatory response [209]. Furthermore, the authors showed that blocking the secretion of EVs could decrease inflammation [209]. Additional studies further demonstrated a potential route of communication between the periphery and the brain parenchyma through EVs. Indeed, plasma exosomes were preferentially taken up by microglia after direct injection into the hippocampus [210]. In the AD mouse model, the uptake of plasma exosomes by microglia was reduced with accumulation around Aβ plaques that are then phagocyted by responsive microglia [210]. Exosomes derived from plasma samples isolated from children with autism spectrum disorder promoted human microglia reactivity in vitro, increasing IL-1β secretion [211]. 

In the retina studies addressing the impact of EVs on microglia are still scarce although a recent report shows that the subretinal administration of exosomes derived from neural progenitor cells could decrease photoreceptor loss in a model of retinal degeneration through the modulation of microglial cell response [212]. The authors demonstrated that these exosomes were selectively incorporated by retinal microglia and suppress the inflammatory signal through the delivery of 17 miRNA targeting TNF, IL-1β and cyclooxygenase-2 [212]. 

## 7. Conclusions

Since their discovery, the role of EVs in diseases onset and progression and their potential as therapeutic agents has been extensively explored. In the beginning regarded as cell waste materials, EVs were later viewed as a mirror of the cell status, but more recently evidence demonstrate that cargo is selectively loaded into EVs, acting as message carriers [213]. These small vesicles were shown to have an enormous potential as disease biomarkers and therapeutic agents [68,69,71,214]. Several studies explored the application EVs to halt disease progression or even engineered them to act as reservoirs for therapeutic agents [215]. Although substantial breakthroughs were made in the field, several drawbacks remain to be surpassed. The different isolation techniques lead to a heterogeneity in EVs populations even when collecting from the same cellular source [214,216]. Indeed, recently the combined use of several techniques to increase EVs purity was proposed [217]. In addition, attempts to purify a specific type of EVs are often unsuccessful, either with a very low yield or presence of contaminants [216]. Recently guidelines for the identification and isolation of EVs have been developed in an attempt to unify the classification of EVs and apply minimal standards for study reporting [73].

Mounting evidence suggests that EVs are of the highest importance in microglial cell function. EVs act as a spreader of information and help orchestrate the response of the cells, functioning as a specific communication strategy either between microglial cells, with the other cells in the parenchyma or even at long distance. Microglia were also found to act as a hub for EVs, shifting their response and signaling to other cells. Despite the promising data compiled herein, more studies performed in vivo and in animal models on the role of microglia EVs are still needed, since most studies were performed in vitro, discarding the contribution of the complex living organism. However, pioneering studies in the field are beginning to explore the use of neuronal EVs as biomarkers for cognitive decline, as reported recently in [218]. EVs isolated from AD patients were shown to induce tau misfolding in interneurons upon brain injection [219]. This may envisage that, in a near future, similar studies could be conducted in other neurodegenerative disorders aiming at exploring alterations in microglial EVs in early disease. Therefore, new strategies that modulate the biogenesis of these vesicles may provide a scientific innovation in diagnostic and therapeutics. Furthermore, new information should be gathered regarding the cargo selection of microglial EVs. Substantial breakthroughs in this field could be achieved by the clear identification of the content of microglial EVs and how it can change in specific pathologies. Indeed, there is no doubt that the modulation of microglia EVs may offer the potential for the design of better and targeted therapies for neurodegenerative disorders. 

## References

[1] Lawson L.J., Perry V.H., Dri P., Gordon S. (1990). Heterogeneity in the distribution and morphology of microglia in the normal adult mouse brain. Neuroscience.

[2] Li F., Jiang D., Samuel M.A. (2019). Microglia in the developing retina. Neural Dev..

[3] Hickman S., Izzy S., Sen P., Morsett L., El Khoury J. (2018). Microglia in neurodegeneration. Nat. Neurosci..

[4] Kettenmann H., Hanisch U.-K., Noda M., Verkhratsky A. (2011). Physiology of Microglia. Physiol. Rev..

[5] Madeira M.H., Boia R., Santos P.F., Ambrósio A.F., Santiago A.R. (2015). Contribution of Microglia-Mediated Neuroinflammation to Retinal Degenerative Diseases. Mediat. Inflamm..

[6] Paolicelli R.C., Bergamini G., Rajendran L. (2019). Cell-to-cell Communication by Extracellular Vesicles: Focus on Microglia. Neuroscience.

[7] Song W.M., Colonna M. (2018). The identity and function of microglia in neurodegeneration. Nat. Immunol..

[8] Hanisch U.-K., Kettenmann H. (2007). Microglia: Active sensor and versatile effector cells in the normal and pathologic brain. Nat. Neurosci..

[9] Itagaki S., McGeer P., Akiyama H., Zhu S., Selkoe D. (1989). Relationship of microglia and astrocytes to amyloid deposits of Alzheimer disease. J. Neuroimmunol..

[10] Lee C.Y.D., Landreth G.E. (2010). The role of microglia in amyloid clearance from the AD brain. J. Neural Transm..

[11] Wegiel J., Imaki H., Wang K.-C., Wegiel J., Wronska A., Osuchowski M., Rubenstein R. (2003). Origin and turnover of microglial cells in fibrillar plaques of APPsw transgenic mice. Acta Neuropathol..

[12] Wegiel J., Imaki H., Wang K.-C., Wegiel J., Rubenstein R. (2004). Cells of monocyte/microglial lineage are involved in both microvessel amyloidosis and fibrillar plaque formation in APPsw tg mice. Brain Res..

[13] Meyer-Luehmann M., Spires-Jones T.L., Prada C., Garcia-Alloza M., de Calignon A., Rozkalne A., Koenigsknecht-Talboo J., Holtzman D.M., Bacskai B.J., Hyman B.T. (2008). Rapid appearance and local toxicity of amyloid-β plaques in a mouse model of Alzheimer’s disease. Nature.

[14] Bolmont T., Haiss F., Eicke D., Radde R., Mathis C.A., Klunk W.E., Kohsaka S., Jucker M., Calhoun M.E. (2008). Dynamics of the Microglial/Amyloid Interaction Indicate a Role in Plaque Maintenance. J. Neurosci..

[15] Paresce D.M., Ghosh R.N., Maxfield F.R. (1996). Microglial Cells Internalize Aggregates of the Alzheimer’s Disease Amyloid β-Protein Via a Scavenger Receptor. Neuron.

[16] Condello C., Yuan P., Schain A., Grutzendler J. (2015). Microglia constitute a barrier that prevents neurotoxic protofibrillar Aβ42 hotspots around plaques. Nat. Commun..

[17] Schmid C.D., Sautkulis L.N., Danielson P.E., Cooper J., Hasel K.W., Hilbush B.S., Sutcliffe J.G., Carson M.J. (2002). Heterogeneous expression of the triggering receptor expressed on myeloid cells-2 on adult murine microglia. J. Neurochem..

[18] Ruiz A., Dols-Icardo O., Bullido M.J., Pastor P., Rodríguez-Rodríguez E., de Munain A.L., Pancorbo M.D.L.A.M.D., Pérez-Tur J., Álvarez V., Antonell A. (2014). Assessing the role of the TREM2 p.R47H variant as a risk factor for Alzheimer’s disease and frontotemporal dementia. Neurobiol. Aging.

[19] Korvatska O., Leverenz J.B., Jayadev S., McMillan P., Kurtz I., Guo X., Rumbaugh M., Matsushita M., Girirajan S., Dorschner M.O. (2015). R47H Variant of TREM2 Associated with Alzheimer Disease in a Large Late-Onset Family: Clinical, Genetic, and Neuropathological Study. JAMA Neurol..

[20] Sobue A., Komine O., Hara Y., Endo F., Mizoguchi H., Watanabe S., Murayama S., Saito T., Saido T.C., Sahara N. (2021). Microglial gene signature reveals loss of homeostatic microglia associated with neurodegeneration of Alzheimer’s disease. Acta Neuropathol. Commun..

[21] Zrzavy T., Hametner S., Wimmer I., Butovsky O., Weiner H.L., Lassmann H. (2017). Loss of ‘homeostatic’ microglia and patterns of their activation in active multiple sclerosis. Brain.

[22] Lassmann H., Brück W., Lucchinetti C.F. (2007). The Immunopathology of Multiple Sclerosis: An Overview. Brain Pathol..

[23] Chen W.-W., Zhang X., Huang W.-J. (2016). Role of neuroinflammation in neurodegenerative diseases (Review). Mol. Med. Rep..

[24] Amor S., Puentes F., Baker D., Van Der Valk P. (2010). Inflammation in neurodegenerative diseases. Immunology.

[25] Hickman S.E., Allison E.K., El Khoury J. (2008). Microglial Dysfunction and Defective β-Amyloid Clearance Pathways in Aging Alzheimer’s Disease Mice. J. Neurosci..

[26] Sveinbjornsdottir S. (2016). The clinical symptoms of Parkinson’s disease. J. Neurochem..

[27] Zhang W., Wang T., Pei Z., Miller D.S., Wu X., Block M.L., Wilson B., Zhang W., Zhou Y., Hong J.-S. (2005). Aggregated α-synuclein activates microglia: A process leading to disease progression in Parkinson’s disease. FASEB J..

[28] Su X., Maguire-Zeiss K.A., Giuliano R., Prifti L., Venkatesh K., Federoff H.J. (2008). Synuclein activates microglia in a model of Parkinson’s disease. Neurobiol. Aging.

[29] Ouchi Y., Yagi S., Yokokura M., Sakamoto M. (2009). Neuroinflammation in the living brain of Parkinson’s disease. Park. Relat. Disord..

[30] McGeer P.L., McGeer P.L. (2007). Glial reactions in Parkinson’s disease. Mov. Disord..

[31] McGeer P.L., Itagaki S., Boyes B.E., McGeer E.G. (1988). Reactive microglia are positive for HLA-DR in the substantia nigra of Parkinson’s and Alzheimer’s disease brains. Neurology.

[32] Gerhard A., Pavese N., Hotton G., Turkheimer F., Es M., Hammers A., Eggert K., Oertel W., Banati R.B., Brooks D.J. (2006). In vivo imaging of microglial activation with [11C](R)-PK11195 PET in idiopathic Parkinson’s disease. Neurobiol. Dis..

[33] Nagatsu T., Mogi M., Ichinose H., Togari A. (2000). Cytokines in Parkinson’s disease. J. Neur. Transmis. Supplementum.

[34] Litteljohn D., Hayley S. (2012). Cytokines as Potential Biomarkers for Parkinson’s Disease: A Multiplex Approach. Adv. Struct. Saf. Stud..

[35] Mount M.P., Lira A., Grimes D., Smith P.D., Faucher S., Slack R., Anisman H., Hayley S., Park D.S. (2007). Involvement of Interferon- in Microglial-Mediated Loss of Dopaminergic Neurons. J. Neurosci..

[36] Zhang D., Li S., Hou L., Jing L., Ruan Z., Peng B., Zhang X., Hong J.-S., Zhao J., Wang Q. (2021). Microglial activation contributes to cognitive impairments in rotenone-induced mouse Parkinson’s disease model. J. Neuroinflamm..

[37] Loane D.J., Kumar A., Stoica B.A., Cabatbat R., Faden A.I. (2014). Progressive Neurodegeneration After Experimental Brain Trauma. J. Neuropathol. Exp. Neurol..

[38] Ramlackhansingh A.F., Brooks D.J., Greenwood R.J., Bose S.K., Turkheimer F.E., Kinnunen K.M., Gentleman S., Heckemann R.A., Gunanayagam K., Gelosa G. (2011). Inflammation after trauma: Microglial activation and traumatic brain injury. Ann. Neurol..

[39] Koshinaga M., Katayama Y., Fukushima M., Oshima H., Suma T., Takahata T. (2000). Rapid and Widespread Microglial Activation Induced by Traumatic Brain Injury in Rat Brain Slices. J. Neurotrauma.

[40] Piao C.-S., Stoica B.A., Wu J., Sabirzhanov B., Zhao Z., Cabatbat R., Loane D.J., Faden A.I. (2013). Late exercise reduces neuroinflammation and cognitive dysfunction after traumatic brain injury. Neurobiol. Dis..

[41] Lin C.-T., Lecca D., Yang L.-Y., Luo W., Scerba M.T., Tweedie D., Huang P.-S., Jung Y.-J., Kim D.S., Yang C.-H. (2020). 3,6′-Dithiopomalidomide reduces neural loss, inflammation, behavioral deficits in brain injury and microglial activation. eLife.

[42] Byrnes K.R., Loane D.J., Stoica B.A., Zhang J., Faden A.I. (2012). Delayed mGluR5 activation limits neuroinflammation and neurodegeneration after traumatic brain injury. J. Neuroinflamm..

[43] Henry R.J., Ritzel R.M., Barrett J.P., Doran S.J., Jiao Y., Leach J.B., Szeto G.L., Wu J., Stoica B.A., Faden A.I. (2020). Microglial Depletion with CSF1R Inhibitor During Chronic Phase of Experimental Traumatic Brain Injury Reduces Neurodegeneration and Neurological Deficits. J. Neurosci..

[44] Langmann T. (2007). Microglia activation in retinal degeneration. J. Leukoc. Biol..

[45] Rathnasamy G., Foulds W.S., Ling E.-A., Kaur C. (2019). Retinal microglia—A key player in healthy and diseased retina. Prog. Neurobiol..

[46] Aires I.D., Ribeiro-Rodrigues T., Boia R., Catarino S., Girão H., Ambrósio A.F., Santiago A.R. (2020). Exosomes derived from microglia exposed to elevated pressure amplify the neuroinflammatory response in retinal cells. Glia.

[47] Xu W., Wu Y., Hu Z., Sun L., Dou G., Zhang Z., Wang H., Guo C., Wang Y. (2019). Exosomes from Microglia Attenuate Photoreceptor Injury and Neovascularization in an Animal Model of Retinopathy of Prematurity. Mol. Ther. Nucleic Acids.

[48] Jutley G., Luk S.M., Dehabadi M.H., Cordeiro M.F. (2017). Management of glaucoma as a neurodegenerative disease. Neurodegener. Dis. Manag..

[49] Gupta N., Yücel Y.H. (2007). Glaucoma as a neurodegenerative disease. Curr. Opin. Ophthalmol..

[50] Shahsuvaryan M.L. (2013). Glaucomatous Optic Neuropathy Management: The Role of Neuroprotective Agents. Med. Hypothesis Discov. Innov. Ophthalmol. J..

[51] Madeira M.H., Boia R., Elvas F., Martins T., Cunha R.A., Ambrósio A.F., Santiago A.R. (2016). Selective A2A receptor antagonist prevents microglia-mediated neuroinflammation and protects retinal ganglion cells from high intraocular pressure–induced transient ischemic injury. Transl. Res..

[52] Aires I.D., Boia R., Rodrigues-Neves A.C., Madeira M.H., Marques C., Ambrósio A.F., Santiago A.R. (2018). Blockade of microglial adenosine A 2A receptor suppresses elevated pressure-induced inflammation, oxidative stress, and cell death in retinal cells. Glia.

[53] Bosco A., Steele M.R., Vetter M.L. (2011). Early microglia activation in a mouse model of chronic glaucoma. J. Comp. Neurol..

[54] (2005). Early Treatment for Retinopathy of Prematurity Cooperative Group The Incidence and Course of Retinopathy of Prematurity: Findings From the Early Treatment for Retinopathy of Prematurity Study. Pediatry.

[55] Rivera J.C., Holm M., Austeng D., Morken T.S., Zhou T. (Ellen), Beaudry-Richard A., Sierra E.M., Dammann O., Chemtob S. (2017). Retinopathy of prematurity: Inflammation, choroidal degeneration, and novel promising therapeutic strategies. J. Neuroinflamm..

[56] Rathi S., Jalali S., Patnaik S., Shahulhameed S., Musada G.R., Balakrishnan D., Rani P.K., Kekunnaya R., Chhablani P.P., Swain S. (2017). Abnormal Complement Activation and Inflammation in the Pathogenesis of Retinopathy of Prematurity. Front. Immunol..

[57] Cocucci E., Racchetti G., Meldolesi J. (2009). Shedding microvesicles: Artefacts no more. Trends Cell Biol..

[58] Budnik V., Ruiz-Cañada C., Wendler V.B.C.R.-C.F. (2016). Extracellular vesicles round off communication in the nervous system. Nat. Rev. Neurosci..

[59] Soria F.N., Pampliega O., Bourdenx M., Meissner W.G., Bezard E., Dehay B. (2017). Exosomes, an Unmasked Culprit in Neurodegenerative Diseases. Front. Neurosci..

[60] Quek C., Hill A.F. (2017). The role of extracellular vesicles in neurodegenerative diseases. Biochem. Biophys. Res. Commun..

[61] Martins-Marques T., Ribeiro-Rodrigues T., De Jager S.C., Zuzarte M., Ferreira C., Cruz P., Reis L., Baptista R., Gonçalves L., Sluijter J.P. (2020). Myocardial infarction affects Cx43 content of extracellular vesicles secreted by cardiomyocytes. Life Sci. Alliance.

[62] Kalluri R. (2016). The biology and function of exosomes in cancer. J. Clin. Investig..

[63] Gao L., Wang L., Dai T., Jin K., Zhang Z., Wang S., Xie F., Fang P., Yang B., Huang H. (2018). Tumor-derived exosomes antagonize innate antiviral immunity. Nat. Immunol..

[64] Altan-Bonnet N. (2016). Extracellular vesicles are the Trojan horses of viral infection. Curr. Opin. Microbiol..

[65] Pathan M., Fonseka P., Chitti S.V., Kang T., Sanwlani R., van Deun J., Hendrix A., Mathivanan S. (2019). Vesiclepedia 2019: A compendium of RNA, proteins, lipids and metabolites in extracellular vesicles. Nucleic Acids Res..

[66] Keerthikumar S., Chisanga D., Ariyaratne D., Al Saffar H., Anand S., Zhao K., Samuel M., Pathan M., Jois M., Chilamkurti N. (2016). ExoCarta: A Web-Based Compendium of Exosomal Cargo. J. Mol. Biol..

[67] Kalluri R., le Bleu V.S. (2020). The biology, function, and biomedical applications of exosomes. Science.

[68] Rizvanov A.A., Shaimardanova A.A., Solovyeva V.V., Chulpanova D.S., James V., Kitaeva K.V. (2020). Extracellular vesicles in the diagnosis and treatment of central nervous system diseases. Neural Regen. Res..

[69] Revenfeld A.L.S., Bæk R., Nielsen M.H., Stensballe A., Varming K., Jørgensen M. (2014). Diagnostic and Prognostic Potential of Extracellular Vesicles in Peripheral Blood. Clin. Ther..

[70] Wiklander O.P.B., Brennan M.Á., Lötvall J., Breakefield X.O., El Andaloussi S. (2019). Advances in therapeutic applications of extracellular vesicles. Sci. Transl. Med..

[71] György B., Hung M.E., Breakefield X.O., Leonard J.N. (2015). Therapeutic Applications of Extracellular Vesicles: Clinical Promise and Open Questions. Annu. Rev. Pharmacol. Toxicol..

[72] Zhang H., Freitas D., Kim H.S., Fabijanic K., Li Z., Chen H., Mark M.T., Molina H., Benito-Martin A., Bojmar L. (2018). Identification of distinct nanoparticles and subsets of extracellular vesicles by asymmetric flow field-flow fractionation. Nat. Cell Biol..

[73] Théry C., Witwer K.W., Aikawa E., Alcaraz M.J., Anderson J.D., Andriantsitohaina R., Antoniou A., Arab T., Archer F., Atkin-Smith G.K. (2018). Minimal information for studies of extracellular vesicles 2018 (MISEV2018): A position statement of the International Society for Extracellular Vesicles and update of the MISEV2014 guidelines. J. Extracell. Vesicles.

[74] Van Niel G., D’Angelo G., Raposo G. (2018). Shedding light on the cell biology of extracellular vesicles. Nat. Rev. Mol. Cell Biol..

[75] Ostrowski M., Carmo N.B., Krumeich S., Fanget I., Raposo G., Savina A., Moita C.F., Schauer K., Hume A., Freitas R.P. (2009). Rab27a and Rab27b control different steps of the exosome secretion pathway. Nat. Cell Biol..

[76] Mathieu M., Martin-Jaular L., Lavieu G., Théry C. (2019). Specificities of secretion and uptake of exosomes and other extracellular vesicles for cell-to-cell communication. Nat. Cell Biol..

[77] Colombo M., Moita C., van Niel G., Kowal J., Vigneron J., Benaroch P., Manel N., Moita L.F., Théry C., Raposo G. (2013). Analysis of ESCRT functions in exosome biogenesis, composition and secretion highlights the heterogeneity of extracellular vesicles. J. Cell Sci..

[78] Baietti M.F., Zhang Z., Mortier E., Melchior A., DeGeest G., Geeraerts A., Ivarsson Y., Depoortere F., Coomans C., Vermeiren E. (2012). Syndecan–syntenin–ALIX regulates the biogenesis of exosomes. Nat. Cell Biol..

[79] Muralidharan-Chari V., Clancy J., Plou C., Romao M., Chavrier P., Raposo G., D’Souza-Schorey C. (2009). ARF6-Regulated Shedding of Tumor Cell-Derived Plasma Membrane Microvesicles. Curr. Biol..

[80] Robbins P.D., Morelli A.E. (2014). Regulation of immune responses by extracellular vesicles. Nat. Rev. Immunol..

[81] Ribeiro-Rodrigues T.M., Laundos T.L., Carvalho R., Almeida D., Pereira R., Coelho-Santos V., Silva A.P., Fernandes R., Zuzarte M., Enguita F.J. (2017). Exosomes secreted by cardiomyocytes subjected to ischaemia promote cardiac angiogenesis. Cardiovasc. Res..

[82] Tian T., Zhu Y.-L., Zhou Y.-Y., Liang G.-F., Wang Y.-Y., Hu F.-H., Xiao Z.-D. (2014). Exosome Uptake through Clathrin-mediated Endocytosis and Macropinocytosis and Mediating miR-21 Delivery. J. Biol. Chem..

[83] Mulcahy L., Pink R.C., Carter D.R.F. (2014). Routes and mechanisms of extracellular vesicle uptake. J. Extracell. Vesicles.

[84] Verdera H.C., Gitz-Francois J.J., Schiffelers R.M., Vader P. (2017). Cellular uptake of extracellular vesicles is mediated by clathrin-independent endocytosis and macropinocytosis. J. Control. Release Soc..

[85] Valadi H., Ekstrom K., Bossios A., Sjostrand M., Lee J.J., Lotvall J.O. (2007). Exosome-mediated transfer of mRNAs and microRNAs is a novel mechanism of genetic exchange between cells. Nat. Cell Biol..

[86] Soares A.R., Martins-Marques T., Ribeiro-Rodrigues T.M., Ferreira J.V., Catarino S., Pinho M.J., Zuzarte M., Anjo S.I., Manadas B., Sluijter J. (2015). Gap junctional protein Cx43 is involved in the communication between extracellular vesicles and mammalian cells. Sci. Rep..

[87] Fitzner D., Schnaars M., van Rossum D., Krishnamoorthy G., Dibaj P., Bakhti M., Regen T., Hanisch U.-K., Simons M. (2011). Selective transfer of exosomes from oligodendrocytes to microglia by macropinocytosis. J. Cell Sci..

[88] Lai C.P., Mardini O., Ericsson M., Prabhakar S., Maguire C.A., Chen J.W., Tannous B.A., Breakefield X.O. (2014). Dynamic Biodistribution of Extracellular Vesicles in Vivo Using a Multimodal Imaging Reporter. ACS Nano.

[89] Hoshino A., Costa-Silva B., Shen T.-L., Rodrigues G., Hashimoto A., Mark M.T., Molina H., Kohsaka S., Di Giannatale A., Ceder S. (2015). Tumour exosome integrins determine organotropic metastasis. Nature.

[90] Gangadaran P., Li X.J., Lee H.W., Oh J.M., Kalimuthu S., Rajendran R.L., Son S.H., Baek S.H., Singh T.D., Zhu L. (2017). A new bioluminescent reporter system to study the biodistribution of systematically injected tumor-derived bioluminescent extracellular vesicles in mice. Oncotarget.

[91] Pitt J.M., Charrier M., Viaud S., André F., Besse B., Chaput N., Zitvogel L. (2014). Dendritic Cell–Derived Exosomes as Immunotherapies in the Fight against Cancer. J. Immunol..

[92] Mendt M., Kamerkar S., Sugimoto H., McAndrews K.M., Wu C.-C., Gagea M., Yang S., Blanko E.V.R., Peng Q., Ma X. (2018). Generation and testing of clinical-grade exosomes for pancreatic cancer. JCI Insight.

[93] Lener T., Gimona M., Aigner L., Börger V., Buzas E., Camussi G., Chaput N., Chatterjee D., Court F.A., Del Portillo H.A. (2015). Applying extracellular vesicles based therapeutics in clinical trials—an ISEV position paper. J. Extracell. Vesicles.

[94] Kamerkar S., le Bleu V.S., Sugimoto H., Yang S., Ruivo C., Melo S.A., Lee J.J., Kalluri R. (2017). Exosomes facilitate therapeutic targeting of oncogenic KRAS in pancreatic cancer. Nat. Cell Biol..

[95] Gangadaran P., Hong C.M., Ahn B.-C. (2018). An Update on in Vivo Imaging of Extracellular Vesicles as Drug Delivery Vehicles. Front. Pharmacol..

[96] EL Andaloussi S., Lakhal S., Mäger I., Wood M.J. (2013). Exosomes for targeted siRNA delivery across biological barriers. Adv. Drug Deliv. Rev..

[97] Agrahari V., Agrahari V., Burnouf P.-A., Chew C.H., Burnouf T. (2019). Extracellular Microvesicles as New Industrial Therapeutic Frontiers. Trends Biotechnol..

[98] Elmore S. (2007). Apoptosis: A review of programmed cell death. Toxicol. Pathol..

[99] Atkin-Smith G.K., Poon I.K. (2017). Disassembly of the Dying: Mechanisms and Functions. Trends Cell Biol..

[100] Tixeira R., Phan T.K., Caruso S., Shi B., Atkin-Smith G.K., Nedeva C., Chow J.D.Y., Puthalakath H., Hulett M.D., Herold M.J. (2020). ROCK1 but not LIMK1 or PAK2 is a key regulator of apoptotic membrane blebbing and cell disassembly. Cell Death Differ..

[101] Coleman M., Sahai E., Yeo M., Bosch M., Dewar A., Olson M.F. (2001). Membrane blebbing during apoptosis results from caspase-mediated activation of ROCK I. Nat. Cell Biol..

[102] Sebbagh M., Renvoizé C., Hamelin J., Riché N., Bertoglio J., Bréard J. (2001). Caspase-3-mediated cleavage of ROCK I induces MLC phosphorylation and apoptotic membrane blebbing. Nat. Cell Biol..

[103] Phan T.K., Ozkocak D.C., Poon I.K.H. (2020). Unleashing the therapeutic potential of apoptotic bodies. Biochem. Soc. Trans..

[104] Atkin-Smith G.K., Miles M.A., Tixeira R., Lay F.T., Duan M., Hawkins C.J., Phan T.K., Paone S., Mathivanan S., Hulett M.D. (2019). Plexin B2 Is a Regulator of Monocyte Apoptotic Cell Disassembly. Cell Rep..

[105] Moss D.K., Betin V.M., Malesinski S.D., Lane J.D. (2006). A novel role for microtubules in apoptotic chromatin dynamics and cellular fragmentation. J. Cell Sci..

[106] Battistelli M., Falcieri E. (2020). Apoptotic Bodies: Particular Extracellular Vesicles Involved in Intercellular Communication. Biology.

[107] Halicka H., Bedner E., Darzynkiewicz Z. (2000). Segregation of RNA and Separate Packaging of DNA and RNA in Apoptotic Bodies during Apoptosis. Exp. Cell Res..

[108] Caruso S., Poon I.K.H. (2018). Apoptotic Cell-Derived Extracellular Vesicles: More Than Just Debris. Front. Immunol..

[109] Holmgren L., Szeles A., Rajnavolgyi E., Folkman J., Klein G., Ernberg I., Falk K.I. (1999). Horizontal transfer of DNA by the uptake of apoptotic bodies. Blood.

[110] Bergsmedh A., Szeles A., Henriksson M., Bratt A., Folkman M.J., Spetz A.-L., Holmgren L. (2001). Horizontal transfer of oncogenes by uptake of apoptotic bodies. Proc. Natl. Acad. Sci. USA.

[111] Zernecke A., Bidzhekov K., Noels H., Shagdarsuren E., Gan L., Denecke B., Hristov M., Köppel T., Jahantigh M.N., Lutgens E. (2009). Delivery of MicroRNA-126 by Apoptotic Bodies Induces CXCL12-Dependent Vascular Protection. Sci. Signal..

[112] Mattson M.P. (2000). Apoptosis in neurodegenerative disorders. Nat. Rev. Mol. Cell Biol..

[113] Neumann H., Kotter M.R., Franklin R. (2008). Debris clearance by microglia: An essential link between degeneration and regeneration. Brain.

[114] Stolzing A., Grune T. (2004). Neuronal apoptotic bodies: Phagocytosis and degradation by primary microglial cells. FASEB J..

[115] Márquez-Ropero M., Benito E., Plaza-Zabala A., Sierra A. (2020). Microglial Corpse Clearance: Lessons from Macrophages. Front. Immunol..

[116] Sierra A., Abiega O., Shahraz A., Neumann H. (2013). Janus-faced microglia: Beneficial and detrimental consequences of microglial phagocytosis. Front. Cell. Neurosci..

[117] Prinz M., Jung S., Priller J. (2019). Microglia Biology: One Century of Evolving Concepts. Cell.

[118] Witting A., Müller P., Herrmann A., Kettenmann H., Nolte C. (2002). Phagocytic Clearance of Apoptotic Neurons by Microglia/Brain Macrophages In Vitro. J. Neurochem..

[119] Yanuck S.F. (2019). Microglial Phagocytosis of Neurons: Diminishing Neuronal Loss in Traumatic, Infectious, Inflammatory, and Autoimmune CNS Disorders. Front. Psychiatry.

[120] De Simone R., Ajmone-Cat M.A., Minghetti L. (2004). Atypical Antiinflammatory Activation of Microglia Induced by Apoptotic Neurons: Possible Role of Phosphatidylserine–Phosphatidylserine Receptor Interaction. Mol. Neurobiol..

[121] Liu B., Wang K., Gao H.-M., Mandavilli B., Wang J.-Y., Hong J.-S. (2001). Molecular consequences of activated microglia in the brain: Overactivation induces apoptosis. J. Neurochem..

[122] Sedgwick A.E., D’Souza-Schorey C. (2018). The biology of extracellular microvesicles. Traffic.

[123] Tricarico C., Clancy J., D’Souza-Schorey C. (2017). Biology and biogenesis of shed microvesicles. Small GTPases.

[124] Leventis P.A., Grinstein S. (2010). The Distribution and Function of Phosphatidylserine in Cellular Membranes. Annu. Rev. Biophys..

[125] Jimenez J.J., Jy W., Mauro L.M., Soderland C., Horstman L.L., Ahn Y.S. (2003). Endothelial cells release phenotypically and quantitatively distinct microparticles in activation and apoptosis. Thromb. Res..

[126] Exner T., Ma D.D.F., Joseph J.E., Connor D.E. (2010). The majority of circulating platelet-derived microparticles fail to bind annexin V, lack phospholipid-dependent procoagulant activity and demonstrate greater expression of glycoprotein Ib. Thromb. Haemost..

[127] Del Conde I., Shrimpton C.N., Thiagarajan P., López J.A. (2005). Tissue-factor–bearing microvesicles arise from lipid rafts and fuse with activated platelets to initiate coagulation. Blood.

[128] Muralidharan-Chari V., Clancy J., Sedgwick A., D’Souza-Schorey C. (2010). Microvesicles: Mediators of extracellular communication during cancer progression. J. Cell Sci..

[129] Tamai K., Tanaka N., Nakano T., Kakazu E., Kondo Y., Inoue J., Shiina M., Fukushima K., Hoshino T., Sano K. (2010). Exosome secretion of dendritic cells is regulated by Hrs, an ESCRT-0 protein. Biochem. Biophys. Res. Commun..

[130] Roucourt B., Meeussen S., Bao J., Zimmermann P., David G. (2015). Heparanase activates the syndecan-syntenin-ALIX exosome pathway. Cell Res..

[131] Henne W.M., Buchkovich N.J., Emr S.D. (2011). The ESCRT Pathway. Dev. Cell.

[132] Schmidt O., Teis D. (2012). The ESCRT machinery. Curr. Biol..

[133] Stuffers S., Wegner C.S., Stenmark H., Brech A. (2009). Multivesicular endosome biogenesis in the absence of ESCRTs. Traffic.

[134] Trajkovic K., Hsu C., Chiantia S., Rajendran L., Wenzel D., Wieland F., Schwille P., Brugger B., Simons M. (2008). Ceramide Triggers Budding of Exosome Vesicles into Multivesicular Endosomes. Science.

[135] Kajimoto T., Okada T., Miya S., Zhang L., Nakamura S.-I. (2013). Ongoing activation of sphingosine 1-phosphate receptors mediates maturation of exosomal multivesicular endosomes. Nat. Commun..

[136] Bianco F., Perrotta C., Novellino L., Francolini M., Riganti L., Menna E., Saglietti L., Schuchman E.H., Furlan R., Clementi E. (2009). Acid sphingomyelinase activity triggers microparticle release from glial cells. EMBO J..

[137] Kowal J., Tkach M., Théry C. (2014). Biogenesis and secretion of exosomes. Curr. Opin. Cell Biol..

[138] Bianco F., Pravettoni E., Colombo A., Schenk U., Möller T., Matteoli M., Verderio C. (2005). Astrocyte-Derived ATP Induces Vesicle Shedding and IL-1β Release from Microglia. J. Immunol..

[139] Li J., Li X., Jiang X., Yang M., Yang R., Burnstock G., Xiang Z., Yuan H. (2016). Microvesicles shed from microglia activated by the P2X7-p38 pathway are involved in neuropathic pain induced by spinal nerve ligation in rats. Purinergic Signal..

[140] Sappington R.M., Calkins D.J. (2008). Contribution of TRPV1 to Microglia-Derived IL-6 and NFκB Translocation with Elevated Hydrostatic Pressure. Investig. Opthalmology Vis. Sci..

[141] Marrone M.C., Morabito A., Giustizieri M., Chiurchiù V., Leuti A., Mattioli M., Marinelli S., Riganti L., Lombardi M., Murana E. (2017). TRPV1 channels are critical brain inflammation detectors and neuropathic pain biomarkers in mice. Nat. Commun..

[142] Kumar A., Stoica B.A., Loane D.J., Yang M., Abulwerdi G., Khan N., Kumar A., Thom S.R., Faden A.I. (2017). Microglial-derived microparticles mediate neuroinflammation after traumatic brain injury. J. Neuroinflamm..

[143] Yang Y., Boza-Serrano A., Dunning C.J.R., Clausen B.H., Lambertsen K.L., Deierborg T. (2018). Inflammation leads to distinct populations of extracellular vesicles from microglia. J. Neuroinflamm..

[144] Verderio C., Muzio L., Turola E., Bergami A., Novellino L., Ruffini F., Riganti L., Corradini I., Francolini M., Garzetti L. (2012). Myeloid microvesicles are a marker and therapeutic target for neuroinflammation. Ann. Neurol..

[145] Joshi P.G., Turola E., Ruiz A., Bergami A., Libera D.D., Benussi L., Giussani P., Magnani G., Comi G., Legname G. (2014). Microglia convert aggregated amyloid-β into neurotoxic forms through the shedding of microvesicles. Cell Death Differ..

[146] Agosta F., Libera D.D., Spinelli E.G., Finardi A., Canu E., Bergami A., Chiavetto L.B., Baronio M., Comi G., Martino G. (2014). Myeloid microvesicles in cerebrospinal fluid are associated with myelin damage and neuronal loss in mild cognitive impairment and Alzheimer disease. Ann. Neurol..

[147] Walsh D.M., Selkoe D.J. (2007). A? Oligomers? a decade of discovery. J. Neurochem..

[148] Prada I., Gabrielli M., Turola E., Iorio A., D’Arrigo G., Parolisi R., De Luca M., Pacifici M., Bastoni M., Lombardi M. (2018). Glia-to-neuron transfer of miRNAs via extracellular vesicles: A new mechanism underlying inflammation-induced synaptic alterations. Acta Neuropathol..

[149] Antonucci F., Turola E., Riganti L., Caleo M., Gabrielli M., Perrotta C., Novellino L., Clementi E., Giussani P., Viani P. (2012). Microvesicles released from microglia stimulate synaptic activity via enhanced sphingolipid metabolism. EMBO J..

[150] Gabrielli M., Battista N., Riganti L., Prada I., Antonucci F., Cantone L., Matteoli M., Maccarrone M., Verderio C. (2015). Active endocannabinoids are secreted on extracellular membrane vesicles. EMBO Rep..

[151] Glebov K., Löchner M., Jabs R., Lau T., Merkel O., Schloss P., Steinhäuser C., Walter J. (2014). Serotonin stimulates secretion of exosomes from microglia cells. Glia.

[152] Drago F., Lombardi M., Prada I., Gabrielli M., Joshi P., Cojoc D., Franck J., Fournier I., Vizioli J., Verderio C. (2017). ATP Modifies the Proteome of Extracellular Vesicles Released by Microglia and Influences Their Action on Astrocytes. Front. Pharmacol..

[153] Potolicchio I., Carven G.J., Xu X., Stipp C., Riese R.J., Stern L.J., Santambrogio L. (2005). Proteomic Analysis of Microglia-Derived Exosomes: Metabolic Role of the Aminopeptidase CD13 in Neuropeptide Catabolism. J. Immunol..

[154] Mukherjee S., Cabrera M.A., Boyadjieva N.I., Berger G., Rousseau B., Sarkar D.K. (2020). Alcohol Increases Exosome Release from Microglia to Promote Complement C1q-Induced Cellular Death of Proopiomelanocortin Neurons in the Hypothalamus in a Rat Model of Fetal Alcohol Spectrum Disorders. J. Neurosci..

[155] Lemaire Q., Raffo-Romero A., Arab T., Van Camp C., Drago F., Forte S., Gimeno J.-P., Begard S., Colin M., Vizioli J. (2019). Isolation of microglia-derived extracellular vesicles: Towards miRNA signatures and neuroprotection. J. Nanobiotechnol..

[156] Tsutsumi R., Hori Y., Seki T., Kurauchi Y., Sato M., Oshima M., Hisatsune A., Katsuki H. (2019). Involvement of exosomes in dopaminergic neurodegeneration by microglial activation in midbrain slice cultures. Biochem. Biophys. Res. Commun..

[157] Takenouchi T., Tsukimoto M., Iwamaru Y., Sugama S., Sekiyama K., Sato M., Kojima S., Hashimoto M., Kitani H. (2015). Extracellular ATP induces unconventional release of glyceraldehyde-3-phosphate dehydrogenase from microglial cells. Immunol. Lett..

[158] Ruan Z., Delpech J.-C., Kalavai S.V., Van Enoo A.A., Hu J., Ikezu S., Ikezu T. (2020). P2RX7 inhibitor suppresses exosome secretion and disease phenotype in P301S tau transgenic mice. Mol. Neurodegener..

[159] Di Virgilio F., dal Ben D., Sarti A.C., Giuliani A.L., Falzoni S. (2017). The P2X7 receptor in infection and inflammation. Immunity.

[160] Chang C., Lang H., Geng N., Wang J., Li N., Wang X. (2013). Exosomes of BV-2 cells induced by alpha-synuclein: Important mediator of neurodegeneration in PD. Neurosci. Lett..

[161] Asai H., Ikezu S., Tsunoda S., Medalla M., Luebke J., Haydar T., Wolozin B., Butovsky O., Kügler S., Ikezu T. (2015). Depletion of microglia and inhibition of exosome synthesis halt tau propagation. Nat. Neurosci..

[162] Zheng T., Wu X., Wei X., Wang M., Zhang B. (2018). The release and transmission of amyloid precursor protein via exosomes. Neurochem. Int..

[163] Yuyama K., Igarashi Y. (2017). Exosomes as Carriers of Alzheimer’s Amyloid-ß. Front. Neurosci..

[164] Sinha M.S., Ansell-Schultz A., Civitelli L., Hildesjö C., Larsson M., Lannfelt L., Ingelsson M., Hallbeck M. (2018). Alzheimer’s disease pathology propagation by exosomes containing toxic amyloid-beta oligomers. Acta Neuropathol..

[165] Malm T., Loppi S., Kanninen K.M. (2016). Exosomes in Alzheimer’s disease. Neurochem. Int..

[166] Laulagnier K., Javalet C., Hemming F.J., Chivet M., Lachenal G., Blot B., Chatellard C., Sadoul R. (2017). Amyloid precursor protein products concentrate in a subset of exosomes specifically endocytosed by neurons. Cell. Mol. Life Sci..

[167] Tofaris G.K. (2017). A Critical Assessment of Exosomes in the Pathogenesis and Stratification of Parkinson’s Disease. J. Park. Dis..

[168] Porro C., Panaro M.A., Lofrumento D.D., Hasalla E., Trotta T. (2019). The multiple roles of exosomes in Parkinson’s disease: An overview. Immunopharmacol. Immunotoxicol..

[169] Yu H., Sun T., An J., Wen L., Liu F., Bu Z., Cui Y., Feng J. (2020). Potential Roles of Exosomes in Parkinson’s Disease: From Pathogenesis, Diagnosis, and Treatment to Prognosis. Front. Cell Dev. Biol..

[170] Xie A., Gao J., Xu L., Meng D. (2014). Shared Mechanisms of Neurodegeneration in Alzheimer’s Disease and Parkinson’s Disease. BioMed. Res. Int..

[171] D’Anca M., Fenoglio C., Serpente M., Arosio B., Cesari M., Scarpini E.A., Galimberti D. (2019). Exosome Determinants of Physiological Aging and Age-Related Neurodegenerative Diseases. Front. Aging Neurosci..

[172] Guo M., Wang J., Zhao Y., Feng Y., Han S., Dong Q., Cui M., Tieu K. (2020). Microglial exosomes facilitate α-synuclein transmission in Parkinson’s disease. Brain.

[173] Braak H., Braak E. (1991). Neuropathological stageing of Alzheimer-related changes. Acta Neuropathol..

[174] Clavaguera F., Bolmont T., Crowther R.A., Abramowski D., Frank S., Probst A., Fraser G., Stalder A.K., Beibel M., Staufenbiel M. (2009). Transmission and spreading of tauopathy in transgenic mouse brain. Nat. Cell Biol..

[175] Lim J., Yue Z. (2015). Neuronal Aggregates: Formation, Clearance, and Spreading. Dev. Cell.

[176] Desdín-Micó G., Mittelbrunn M. (2017). Role of exosomes in the protection of cellular homeostasis. Cell Adhes. Migr..

[177] Emedina M., Avila J. (2014). The role of extracellular Tau in the spreading of neurofibrillary pathology. Front. Cell. Neurosci..

[178] Chivet M., Javalet C., Hemming F., Laulagnier K., Fraboulet S., Sadoul R., Pernet-Gallay K. (2013). Exosomes as a novel way of interneuronal communication. Biochem. Soc. Trans..

[179] Crotti A., Sait H.R., McAvoy K.M., Estrada K., Ergun A., Szak S., Marsh G., Jandreski L., Peterson M., Reynolds T.L. (2019). BIN1 favors the spreading of Tau via extracellular vesicles. Sci. Rep..

[180] Clayton K., Delpech J.C., Herron S., Iwahara N., Ericsson M., Saito T., Saido T.C., Ikezu S., Ikezu T. (2021). Plaque associated microglia hyper-secrete extracellular vesicles and accelerate tau propagation in a humanized APP mouse model. Mol. Neurodegener..

[181] Dinkins M.B., Dasgupta S., Wang G., Zhu G., Bieberich E. (2014). Exosome reduction in vivo is associated with lower amyloid plaque load in the 5XFAD mouse model of Alzheimer’s disease. Neurobiol. Aging.

[182] Tamboli I.Y., Barth E., Christian L., Siepmann M., Kumar S., Singh S., Tolksdorf K., Heneka M.T., Lütjohann D., Wunderlich P. (2010). Statins Promote the Degradation of Extracellular Amyloid β-Peptide by Microglia via Stimulation of Exosome-associated Insulin-degrading Enzyme (IDE) Secretion*. J. Biol. Chem..

[183] Hooper C., Sainz-Fuertes R., Lynham S., Hye A., Killick R., Warley A., Bolondi C., Pocock J., Lovestone S. (2012). Wnt3a induces exosome secretion from primary cultured rat microglia. BMC Neurosci..

[184] Gao G., Zhao S., Xia X., Li C., Li C., Ji C., Sheng S., Tang Y., Zhu J., Wang Y. (2019). Glutaminase C Regulates Microglial Activation and Pro-inflammatory Exosome Release: Relevance to the Pathogenesis of Alzheimer’s Disease. Front. Cell. Neurosci..

[185] Block M.L., Hong J.-S. (2005). Microglia and inflammation-mediated neurodegeneration: Multiple triggers with a common mechanism. Prog. Neurobiol..

[186] Huang Y., Zhao L., Jia B., Wu L., Li Y., Curthoys N., Zheng J.C. (2011). Glutaminase Dysregulation in HIV-1-Infected Human Microglia Mediates Neurotoxicity: Relevant to HIV-1-Associated Neurocognitive Disorders. J. Neurosci..

[187] Gao G., Li C., Zhu J., Wang Y., Huang Y., Zhao S., Sheng S., Song Y., Ji C., Li C. (2020). Glutaminase 1 Regulates Neuroinflammation After Cerebral Ischemia Through Enhancing Microglial Activation and Pro-Inflammatory Exosome Release. Front. Immunol..

[188] Curthoys N.P., Watford M. (1995). Regulation of Glutaminase Activity and Glutamine Metabolism. Annu. Rev. Nutr..

[189] Xie L., Zhao H., Wang Y., Chen Z. (2020). Exosomal shuttled miR-424-5p from ischemic preconditioned microglia mediates cerebral endothelial cell injury through negatively regulation of FGF2/STAT3 pathway. Exp. Neurol..

[190] Boia R., Ruzafa N., Aires I.D., Pereiro X., Ambrósio A.F., Vecino E., Santiago A.R. (2020). Neuroprotective Strategies for Retinal Ganglion Cell Degeneration: Current Status and Challenges Ahead. Int. J. Mol. Sci..

[191] Madeira M.H., Elvas F., Boia R., Gonçalves F.Q., Cunha R.A., Ambrósio A.F., Santiago A.R. (2015). Adenosine A2AR blockade prevents neuroinflammation-induced death of retinal ganglion cells caused by elevated pressure. J. Neuroinflamm..

[192] Madeira M.H., Ortin-Martinez A., Nadal-Nicolás F.M., Ambrósio A.F., Vidal-Sanz M., Agudo-Barriuso M., Santiago A.R. (2016). Caffeine administration prevents retinal neuroinflammation and loss of retinal ganglion cells in an animal model of glaucoma. Sci. Rep..

[193] Yuyama K., Sun H., Mitsutake S., Igarashi Y. (2012). Sphingolipid-modulated Exosome Secretion Promotes Clearance of Amyloid-β by Microglia. J. Biol. Chem..

[194] Yuyama K., Sun H., Sakai S., Mitsutake S., Okada M., Tahara H., Furukawa J.-I., Fujitani N., Shinohara Y., Igarashi Y. (2014). Decreased Amyloid-β Pathologies by Intracerebral Loading of Glycosphingolipid-enriched Exosomes in Alzheimer Model Mice. J. Biol. Chem..

[195] Miyanishi M., Tada K., Koike M., Uchiyama Y., Kitamura T., Nagata S. (2007). Identification of Tim4 as a phosphatidylserine receptor. Nat. Cell Biol..

[196] Skotland T., Hessvik N.P., Sandvig K., Llorente A. (2019). Exosomal lipid composition and the role of ether lipids and phosphoinositides in exosome biology. J. Lipid Res..

[197] Silverman J.M., Fernando S.M., Grad L.I., Hill A.F., Turner B.J., Yerbury J.J., Cashman N.R. (2016). Disease Mechanisms in ALS: Misfolded SOD1 Transferred Through Exosome-Dependent and Exosome-Independent Pathways. Cell. Mol. Neurobiol..

[198] Pinto S., Cunha C., Barbosa M., Vaz A.R., Brites D. (2017). Exosomes from NSC-34 Cells Transfected with hSOD1-G93A Are Enriched in miR-124 and Drive Alterations in Microglia Phenotype. Front. Neurosci..

[199] Fauré J., Lachenal G., Court M., Hirrlinger J., Chatellard-Causse C., Blot B., Grange J., Schoehn G., Goldberg Y., Boyer V. (2006). Exosomes are released by cultured cortical neurones. Mol. Cell. Neurosci..

[200] Bahrini I., Song J.-H., Diez D., Hanayama R. (2015). Neuronal exosomes facilitate synaptic pruning by up-regulating complement factors in microglia. Sci. Rep..

[201] Guo Y., Ji X., Liu J., Fan D., Zhou Q., Chen C., Wang W., Wang G., Wang H., Yuan W. (2019). Effects of exosomes on pre-metastatic niche formation in tumors. Mol. Cancer.

[202] Becker A., Thakur B.K., Weiss J.M., Kim H.S., Peinado H., Lyden D. (2016). Extracellular Vesicles in Cancer: Cell-to-Cell Mediators of Metastasis. Cancer Cell.

[203] De Vrij J., Maas S.N., Kwappenberg K.M., Schnoor R., Kleijn A., Dekker L., Luider T.M., De Witte L.D., Litjens M., Van Strien M.E. (2015). Glioblastoma-derived extracellular vesicles modify the phenotype of monocytic cells. Int. J. Cancer.

[204] Van Der Vos K.E., Abels E.R., Zhang X., Lai C.P.-K., Carrizosa E., Oakley D., Prabhakar S., Mardini O., Crommentuijn M.H.W., Skog J. (2016). Directly visualized glioblastoma-derived extracellular vesicles transfer RNA to microglia/macrophages in the brain. Neuro Oncol..

[205] Cui G., Yan-Wu X., Mou F., Xie W., Wang F., Wang Q., Fang-Fang M., Xu Y., Dong Y., Liu J. (2018). Exosomes derived from hypoxia-preconditioned mesenchymal stromal cells ameliorate cognitive decline by rescuing synaptic dysfunction and regulating inflammatory responses in APP/PS1 mice. FASEB J..

[206] Li Y., Yang Y.-Y., Ren J.-L., Xu F., Chen F.-M., Li A. (2017). Exosomes secreted by stem cells from human exfoliated deciduous teeth contribute to functional recovery after traumatic brain injury by shifting microglia M1/M2 polarization in rats. Stem Cell Res. Ther..

[207] Huang S., Ge X., Zhenyu Y., Han Z., Yin Z., Zhaoli H., Chen F., Wang H., Zhang J., Lei P. (2018). Increased miR-124-3p in microglial exosomes following traumatic brain injury inhibits neuronal inflammation and contributes to neurite outgrowthviatheir transfer into neurons. FASEB J..

[208] Li J.J., Wang B., Kodali M.C., Chen C., Kim E., Patters B.J., Lan L., Kumar S., Wang X., Yue J. (2018). In vivo evidence for the contribution of peripheral circulating inflammatory exosomes to neuroinflammation. J. Neuroinflamm..

[209] Balusu S., Van Wonterghem E., de Rycke R., Raemdonck K., Stremersch S., Gevaert K., Brkic M., Demeestere D., Vanhooren V., Hendrix A. (2016). Identification of a novel mechanism of blood–brain communication during peripheral inflammation via choroid plexus-derived extracellular vesicles. EMBO Mol. Med..

[210] Zheng T., Pu J., Chen Y., Mao Y., Guo Z., Pan H., Zhang L., Zhang H., Sun B., Zhang B. (2017). Plasma Exosomes Spread and Cluster Around β-Amyloid Plaques in an Animal Model of Alzheimer’s Disease. Front. Aging Neurosci..

[211] Tsilioni I., Theoharides T.C. (2018). Extracellular vesicles are increased in the serum of children with autism spectrum disorder, contain mitochondrial DNA, and stimulate human microglia to secrete IL-1β. J. Neuroinflamm..

[212] Bian B., Zhao C., He X., Gong Y., Ren C., Ge L., Zeng Y., Li Q., Chen M., Weng C. (2020). Exosomes derived from neural progenitor cells preserve photoreceptors during retinal degeneration by inactivating microglia. J. Extracell. Vesicles.

[213] Colombo M., Raposo G., Théry C. (2014). Biogenesis, secretion, and intercellular interactions of exosomes and other extracellular vesicles. Annu. Rev. Cell Dev. Biol..

[214] Yang D., Zhang W., Zhang H., Zhang F., Chen L., Ma L., Larcher L., Chen S., Liu N., Zhao Q. (2020). Progress, opportunity, and perspective on exosome isolation—Efforts for efficient exosome-based theranostics. Theranostics.

[215] Kwon S., Shin S., Do M., Oh B.H., Song Y., Bui V.D., Lee E.S., Jo D.-G., Cho Y.W., Kim D.-H. (2021). Engineering approaches for effective therapeutic applications based on extracellular vesicles. J. Control. Release.

[216] Kowal J., Arras G., Colombo M., Jouve M., Morath J.P., Primdal-Bengtson B., Dingli F., Loew D., Tkach M., Théry C. (2016). Proteomic comparison defines novel markers to characterize heterogeneous populations of extracellular vesicle subtypes. Proc. Natl. Acad. Sci. USA.

[217] Stam J., Bartel S., Bischoff R., Wolters J.C. (2021). Isolation of extracellular vesicles with combined enrichment methods. J. Chromatogr. B.

[218] Eren E., Hunt J.F.V., Shardell M., Chawla S., Tran J., Gu J., Vogt N.M., Johnson S.C., Bendlin B.B., Kapogiannis D. (2020). Extracellular vesicle biomarkers of Alzheimer’s disease associated with sub-clinical cognitive decline in late middle age. Alzheimer’s Dement..

[219] Ruan Z., Pathak D., Kalavai S.V., Yoshii-Kitahara A., Muraoka S., Bhatt N., Takamatsu-Yukawa K., Hu J., Wang Y., Hersh S. (2021). Alzheimer’s disease brain-derived extracellular vesicles spread tau pathology in interneurons. Brain.

